# Image quality comparison of synthesized two‐dimensional images and digital mammography in breast tomosynthesis: A quantitative analysis

**DOI:** 10.1002/acm2.70574

**Published:** 2026-04-09

**Authors:** Kazumi Sogabe, Sho Maruyama, Nao Koyama, Hiroki Saito

**Affiliations:** ^1^ Department of Radiological Sciences, School of Health Sciences Ibaraki Prefectural University of Health Sciences Ami Ibaraki Japan; ^2^ Department of Radiological Technology Gunma Prefectural College of Health Sciences Maebashi Gunma Japan; ^3^ Department of Radiology Teikyo University Hospital Itabashi Tokyo Japan; ^4^ Department of Medical Radiology, Faculty of Medical Technology Teikyo University Itabashi Tokyo Japan

**Keywords:** breast cancer, conventional digital mammography, synthesized two‐dimensional mammography

## Abstract

**Background:**

Breast cancer remains a leading cause of cancer‐related morbidity and mortality worldwide. Early detection of breast cancer using high‐quality mammography is essential for improving prognosis and treatment outcomes. Digital breast tomosynthesis with synthesized two‐dimensional mammography (SM) reduces dose and tissue overlap; however, its comprehensive physical evaluations remain limited.

**Purpose:**

This study quantitatively compared the image quality of conventional digital mammography (DM) and SM acquired using the AMULET Innovality System.

**Methods:**

Polymethyl methacrylate, American College of Radiology, and contrast‐detail (CD) mammography phantoms were imaged 10 times in both DM and SM modes. Image quality metrics, including signal‐to‐noise ratio (SNR) maps, noise power spectrum (NPS), contrast‐to‐noise ratio (CNR), modulation transfer function (MTF), and CD curves, were evaluated.

**Results:**

DM demonstrated higher SNR uniformity and suppressed low‐ and high‐frequency noise, and the CNR values were 1.3–2 times greater than those of SM across all object sizes. MTF analysis showed superior resolution in DM (MTF(f50), 4.0 cycles/mm) compared with that of the SM (2.5 cycles/mm). CD curve analysis confirmed the better detectability of fine structures in DM, with $IQF_{inv}$ values of 134.9 for DM and 56.7 for SM.

**Conclusions:**

Quantitative phantom‐based evaluation demonstrated that DM showed superior performance to SM in several physical image quality metrics, including SNR uniformity, noise characteristics, spatial resolution, and contrast–detail detectability. These findings indicate systematic differences in physical image quality between DM and SM; however, further observer and clinical studies are required to clarify how these differences translate to diagnostic performance in clinical practice.

## 1 INTRODUCTION

Breast cancer is a leading cause of cancer‐related morbidity and mortality in women worldwide.^1^, ^2^, ^3^ Early detection of breast cancer using high‐quality mammography is critical for improving prognosis and treatment outcomes.^4^, ^5^ Digital breast tomosynthesis (DBT) was developed to address the limitations of conventional two‐dimensional (2D) digital mammography (DM), particularly tissue overlap, which may obscure lesions or mimic malignancies. DBT acquires projection images from multiple angles and reconstructs thin slices, providing quasi‐three‐dimensional visualization of the breast.^6^ Recently, in DBT‐DM combined protocols, synthetic 2D images (SM) reconstructed from DBT data have been introduced as a substitute for standard DM, enabling the reduction of radiation dose and compression time while maintaining diagnostic performance.^7^ This approach follows the As Low As Reasonably Achievable principle, which is particularly relevant for population‐based screening programs.

Although clinical studies have shown that SM images are not inferior to DM images for lesion detection and breast density assessment, comprehensive physical image quality evaluation remains limited, particularly in systems that use new reconstruction algorithms.^8^ One such system is the AMULET Innovality (Fujifilm, Tokyo, Japan), a DBT platform that incorporates an advanced amorphous selenium detector and vendor‐specific image synthesis techniques. Previous studies have reported certain aspects of image quality for this system.^9^, ^10^ However, to date, no studies have investigated signal‐to‐noise ratio (SNR) maps for SM or DM images generated by AMULET Innovality. As the SNR distribution is instrumental in the visibility of low‐contrast lesions in heterogeneous breast tissue, the absence of such an analysis represents a significant gap in knowledge. Furthermore, visual image quality assessment is subject to observer variability and may be affected over time by hardware aging, calibration changes, or software updates, highlighting the importance of objective and reproducible metrics.^11^, ^12^

This study performed a comprehensive physical image quality evaluation of the AMULET Innovality Tomosynthesis System by comparing conventional DM images with SM images using standardized contrast‐detail (CD) mammography (CDMAM), American College of Radiology (ACR), and polymethyl methacrylate (PMMA) phantoms. Objective image quality parameters, including SNR maps, noise power spectrum (NPS), contrast‐to‐noise ratio (CNR), modulation transfer function (MTF), and CD curves, were measured to quantify and compare the performance across imaging modes. To the best of our knowledge, this is the first study to apply SNR mapping to the AMULET Innovality System, thereby filling a critical gap in the current literature. These findings support the optimization of quality assurance protocols and guide the effective use of synthetic 2D imaging for breast cancer screening and diagnosis.

## 2 MATERIALS AND METHODS

### 2.1 Equipment and phantoms

This study compared the image characteristics of conventional 2D DM and synthesized 2D mammography (SM) using the Fujifilm AMULET Innovality System. Three phantoms were used: a PMMA phantom, an ACR (model‐156) phantom, and a CDMAM phantom. Each phantom was imaged 10 times in both DM and SM modes. For SM acquisition, tomosynthesis was performed in high‐resolution mode.

The NPS, SNR map, and MTF measurements were performed using a PMMA phantom (30 × 24 × 4 cm^3^). For the MTF measurement, a 1‐mm tungsten plate was used in combination with the PMMA phantom. For the ACR phantom, the image analysis focused on tumor‐mimicking objects (diameters of 2.0, 1.0, 0.75, and 0.50 cm), and the CNR was calculated for each region.

To obtain the CD curve, a CDMAM 3.4 phantom (Artinis Medical Systems, Elst, The Netherlands) was used. The phantom was sandwiched between two 2‐cm‐thick PMMA plates, and 10 images were acquired in each DM and SM modes. The phantom position shifted slightly between acquisitions. The images were analyzed using the “CD analysis” ImageJ‐compatible plugin provided by the European Reference Organization for Quality Assured Breast Screening and Diagnostic Services (EUREF), and CD curves were generated.

All images were acquired using automatic exposure control (AEC). The phantoms were positioned according to international guidelines. Image analysis was performed by extracting predefined regions of interest (ROIs) from the acquired images, and the corresponding physical evaluation parameters were calculated.

### 2.2 Image analysis

#### 2.2.1 SNR map

A PMMA phantom (4 cm thick; 30 × 24 cm^2^) was placed at the chest wall edge at the center of the detector, and 10 images were acquired using identical settings. To avoid boundary and edge‐enhancement effects inherent to tomosynthesis reconstruction, a 1‐cm margin from each PMMA edge was excluded, resulting in an analysis region of 4000 × 3200 pixels.

For the 10 images, pixelwise mean and standard deviation (SD) maps were calculated. Specifically, for each pixel location, the pixel values from the 10 images were summed and divided by the number of images to generate the mean map. The SD map was then calculated on a pixel‐by‐pixel basis using the corresponding mean value at each pixel location. Pixelwise SNR maps were generated as the ratio of the mean map to the SD map. The resulting SNR maps were normalized to their maximum value and visualized as color‐coded maps, where the maximum SNR value was displayed in blue, to facilitate visual comparison between DM and SM. All image analyses were performed using Python version 3.9.12 (Python Software Foundation, USA).

To enable a quantitative comparison of SNR maps between SM and DM, the previously defined ROI was subdivided into 1280 non‐overlapping blocks, each consisting of 100 × 100 pixels. The nonuniformity index (NUI) was then calculated from these block‐wise SNR values, following the method described in Ref.^8^ The mean SNR value was calculated for each block, and the NUI was then derived from these block‐wise SNR values to quantify spatial nonuniformity in the SNR map. Thus, the NUI was applied to the SNR map itself, rather than to the original image intensity values.

(1) $$NUI=\frac{\max\left({\bar{PV}}_{ROI_{i}}\right)-\;\min\left({\bar{PV}}_{ROI_{i}}\right)}{\left[\frac{\max\left({\bar{PV}}_{ROI_{i}}\right)+\min\left({\bar{PV}}_{ROI_{i}}\right)}{2}\right]}$$

Here, ${\bar{PV}}_{ROI_{i}}$ denotes the mean pixel value of the *i*‐th ROI, representing the regions with the maximum and minimum average pixel values. As the pixel size of DM is 50 µm and that of SM is 100 µm, the ROI dimensions in SM were set to one‐half of those in DM.

#### 2.2.2 NPS

The same image set used for SNR map analysis was applied to the NPS calculation. An ROI of 128 × 128 pixels was selected at the center of the PMMA phantom. Noised images were generated by subtracting two images acquired under identical conditions. A fast Fourier transform (FFT) was then applied, and the NPS was calculated using Equation (2). The final 2D NPS was obtained by averaging the results from the 10 noise images.

(2) $$NPS\;\left(f_{x},\;f_{y}\right)=\frac{\Delta x\cdot \Delta y}{Nx\cdot \;Ny}\;\left[\lceil FFT\left(ROI_{n}\left(x,\;y\right)\right)\rceil^{2}\right]$$

Here, Δx and Δy represent the pixel sizes, whereas Nx and Ny denote the number of pixels in each direction within the ROI (128 × 128). $ROI_{n}\left(x,\;y\right)$ refers to the “noise” image obtained by subtracting two images acquired under identical conditions. A scaling factor of 1/2 was applied to the obtained results to account for the subtraction operation, which doubles the noise variance.

To further highlight the characteristics of the NPS, the 2D NPS was averaged in the radial direction along the *x*‐(horizontal) and *y*‐axes (vertical).

#### 2.2.3 CNR

For the ACR 156 phantom, the CNR was calculated for each mass object size using Equation (3).

(3) $$CNR=\frac{{\bar{PV}}_{signal}-{\bar{PV}}_{background}\;}{\sigma_{background}}$$

Here, ${\bar{PV}}_{signal}$ represents the mean pixel value inside the mass, and ${\bar{PV}}_{background}$ represents the mean pixel value of a 300 × 300 pixel region containing the mass, with the mass pixels excluded. The background ROI corresponded to a 300 × 300 pixel region centered on the mass, which is the same region shown in the representative result images. Pixels belonging to the mass were excluded from this region by masking the mass area prior to calculation of the background mean and standard deviation. $\sigma_{background}$ denotes the SD within the background region. This local background definition was adopted to minimize the influence of edge enhancement and halo effects observed in synthesized mammography images, which could otherwise bias CNR estimation when using a separate background ROI. Image analysis was performed using ImageJ version 1.54f (National Institutes of Health, USA).

#### 2.2.4 MTF

A 1‐mm tungsten plate was placed at the center of a support platform sandwiched between two 2‐cm‐thick PMMA plates to replicate clinical breast imaging conditions, accounting for attenuation and scatter effects present in clinical practice. The edge was oriented with a small slanted angle relative to the measurement direction (*x*‐ and *y*‐axes). The actual angle was determined directly from the acquired images using linear regression of the detected edge pixels and was 0.97 ± 0.02° and 1.54 ± 0.01° for DM_*x* and DM_*y*, and 0.94 ± 0.07° and 1.54 ± 0.02° for SM_*x* and SM_*y*, respectively. Ten images were acquired under identical conditions in the DM and SM modes.

Composite edge‐spread functions (ESFs) were obtained from the edge images (DM, 256 × 256 pixels; SM, 128 × 128 pixels) by aligning and averaging multiple line profiles perpendicular to the edge direction. Under these reduced‐contrast conditions caused by the presence of PMMA, the composite ESF was modeled using an error function to obtain a stable estimate of the line‐spread function (LSF).^13^ The LSF was derived by differentiating the fitted ESF and subsequently processed using FFT to derive the MTF. Image analysis was performed using Python version 3.9.12.

#### 2.2.5 CD curve

A CDMAM type 3.4 phantom was positioned at the chest wall edge of the support platform and sandwiched between two 2‐cm‐thick PMMA plates. The phantom was shifted slightly for each acquisition to minimize the influence of fixed‐pattern noise and structured background effects, following the methodology described in Refs. 8,10 images were obtained in both DM and SM modes.

Image analysis was performed using the “CD analysis” tool included in the QC_MAM ImageJ plugin distributed by the EUREF.^14^ The CD curve was generated by averaging the fitted threshold gold thickness (µm) values obtained from the 10 images. The inverse image quality figure ($IQF_{inv}$) was then calculated according to Equation (4), which is widely used in CDMAM phantom analysis to summarize contrast–detail detectability into a single index.^15^, ^16^ The $IQF_{inv}$ reflects the combined performance of contrast resolution and spatial resolution, with higher values indicating improved detectability of smaller and lower‐contrast details. This metric was originally introduced for objective evaluation of digital mammography systems and has been widely adopted in CDMAM‐based image quality assessment.

(4) $$IQF_{inv}=\frac{100}{\sum_{i=1}^{16}C_{i}\;\times \;D_{i,\;min}}$$

Here, $C_{i}$ represents the gold disk thickness identified in contrast column *i*, and $D_{i,\;min}$ denotes the threshold diameter of the column. As the threshold thickness decreased, the denominator value decreased, resulting in an increase in the $IQF_{inv}$ value, corresponding to superior overall image quality. Detection probability in the CDMAM analysis was defined as the threshold at which a given gold disk contrast and diameter could be detected with a predefined acceptance rate. Consistent with established CDMAM methodology, a detection probability of 62.5% was used to determine the threshold diameter. Specifically, for each contrast level, the smallest gold disk diameter detected in at least 62.5% of image presentations was recorded as the detection threshold.^17^

## 3 RESULTS

### 3.1 Imaging conditions

Table 1 summarizes the acquisition parameters obtained from the DICOM headers in each experiment. Phantoms were positioned to simulate clinical conditions, and all examinations were performed using AEC.

**TABLE 1:** Exposure parameters used for each experiment under automatic exposure control (AEC) in digital mammography (DM) and synthesized mammography (SM).

			Tube voltage (kV)		Tube current (mA)		Exposure time (ms)	
Test	Pantom	Image	Average	SD	Average	SD	Average	SD
SNR(NPS)	PMMA	DM	29.0	0	79.2	2.71	808.1	27.36
		SM	30.1	0.30	75.5	2.91	630.2	13.56
CNR	ACR156	DM	28.0	0	84.9	2.85	782.6	20.53
		SM	31.0	0	76.5	3.01	609.7	4.69
MTF	1‐mm tungsten plate + PMMA	DM	29.0	0	79.1	2.00	809.3	21.90
		SM	29.2	0.36	74.0	4.11	580.9	3.75
CD curve	CDMAM + PMMA	DM	29.0	0	103.7	1.27	853.5	11.77
		SM	32.0	0	76.6	3.80	696.3	3.55

All measurements were repeated 10 times, and the mean ± standard deviation (SD) is reported to demonstrate the reproducibility and limited variability of exposure conditions under AEC operation. The “Phantom” column indicates the phantom used for each evaluation; “+PMMA” denotes that the phantom was sandwiched between PMMA slabs to simulate clinical imaging conditions.

### 3.2 Image evaluation

#### 3.2.1 SNR map

**FIGURE 1:**
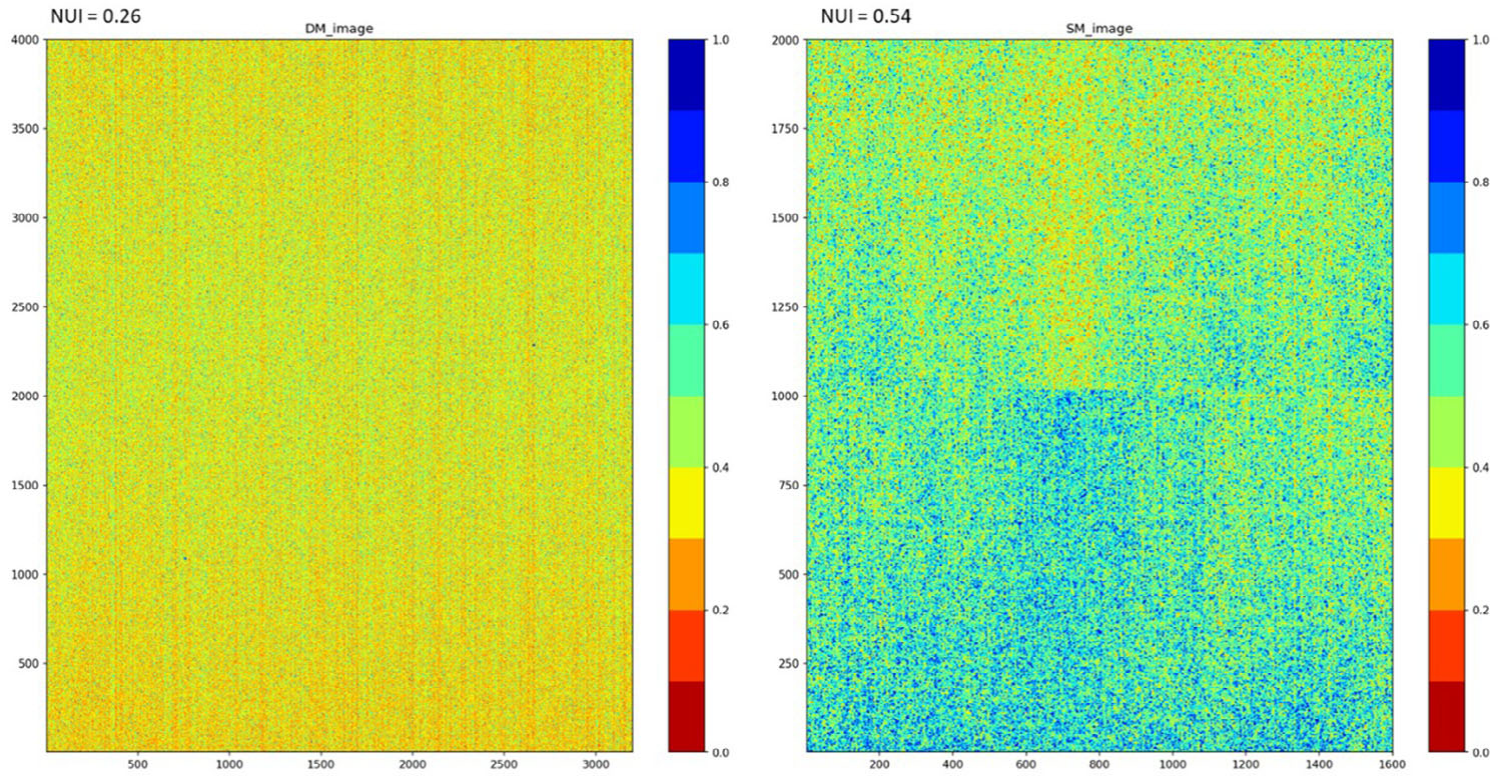
Signal‐to‐noise ratio (SNR) maps for digital mammography (DM) and synthesized mammography (SM). SNR maps were calculated pixelwise as the ratio of the mean image to the standard deviation image obtained from 10 repeated acquisitions using a PMMA phantom. All SNR maps were normalized to their respective maximum values and displayed using the same color scale, with the maximum value indicated in blue. DM showed higher SNR values in the central region, whereas SM exhibited higher values in the inferior region. The corresponding nonuniformity index (NUI) values were 0.26 for DM and 0.54 for SM.

Figure 1 shows the SNR maps for DM and SM. A 4000 × 3200 pixel region was selected within the PMMA phantom from the full image matrix (5928 × 4728 pixels), excluding a 1‐cm margin from the edges. (For SM, the effective matrix size was half that of DM because of its pixel size of 100 µm.) Pixel intensity values are displayed using a color map, with the maximum value represented in blue (1.0). The coordinate origin (0, 0) is located at the lower left of the image and corresponds to the right side of the support platform.

For DM, higher SNR values were observed near the center of the phantom, whereas for SM, elevated SNR values were regionally distributed in the lower portion of the image. The NUIs were 0.26 for DM and 0.54 for SM, indicating greater SNR nonuniformity in SM than in DM.

#### 3.2.2 NPS

**FIGURE 2:**
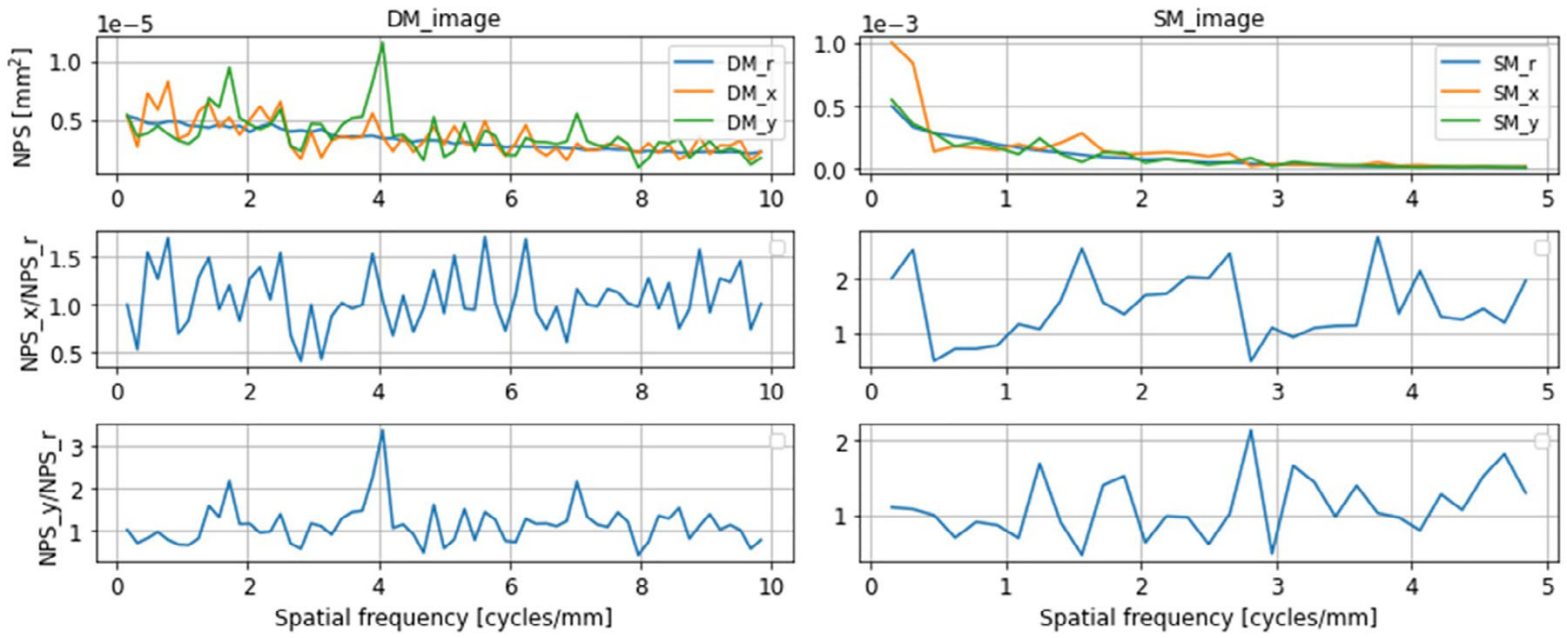
Radial and directional noise power spectra (NPS) for DM and SM. “_r”, “_x”, and “_y” denote the radial, *x*‐axis, and *y*‐axis directions, respectively. Anisotropy ratios were defined as NPS_x/NPS_r and NPS_y/NPS_r. No distinct peaks or periodic structures were observed in the radial NPS. Directional NPS showed non‐systematic fluctuations across spatial frequencies. Anisotropy ratios indicated no significant directional dependence in DM (*p* > 0.05), whereas SM exhibited higher anisotropy along the *x*‐axis (*p* < 0.05).

Figure 2 presents the NPS of DM and SM in the radial, *x*‐, and *y*‐axis directions. The radial component reflects the isotropy of the noise distribution, whereas the *x*‐ and *y*‐axis components indicate anisotropy in their respective directions.

Although DM appeared to exhibit larger fluctuations than SM, the amplitude of the DM NPS was of the order of 10^−5^, which was approximately 1/100 smaller than that of SM. No distinct peaks were observed in the radial NPS of either DM or SM. Directional NPS analysis showed only non‐systematic fluctuations across spatial frequencies, with no evidence of consistent periodic structures. However, anisotropy indices, expressed as NPS_x/NPS_r and NPS_y/NPS_r, which quantify directional differences in overall noise power rather than spectral periodicity, showed no significant difference between directions in DM (*p* > 0.05). In contrast, SM demonstrated higher anisotropy along NPS_x/NPS_r (*p* < 0.05).

#### 3.2.3 CNR

**FIGURE 3:**
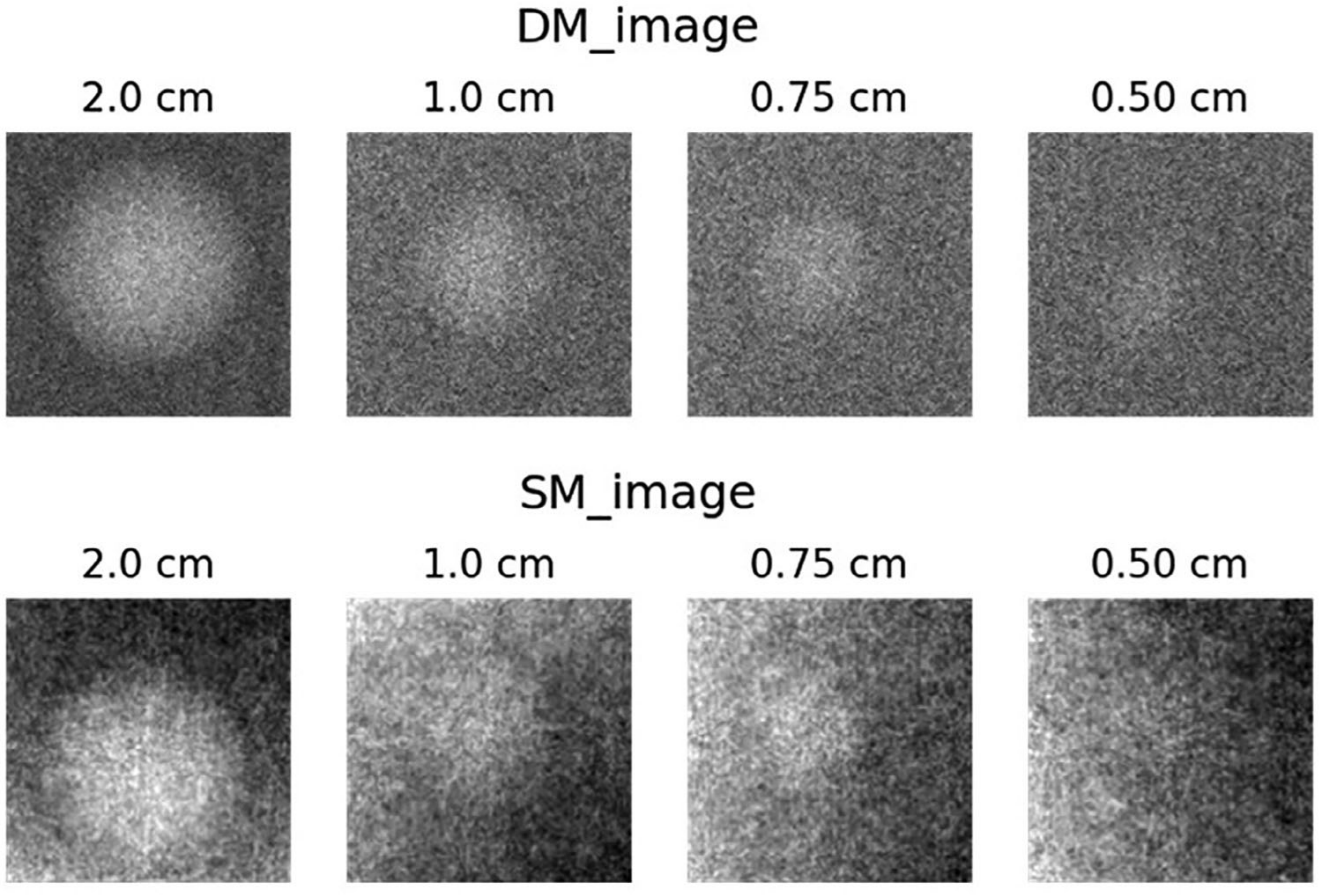
Contrast‐to‐noise ratio (CNR) measurements for mass objects in the ACR phantom for DM and SM. For each mass object size, a 300 × 300 pixel local region of interest (ROI) containing the mass was defined and segmented into the mass region and the surrounding background. Pixels corresponding to the mass were excluded from the background region for CNR calculation. This local background definition was adopted to minimize the influence of edge enhancement and halo effects observed in synthesized mammography images. The measured CNR values were consistently higher in DM than in SM across all mass object sizes.

Figure 3 shows the representative images of the mass objects, and Table 2 summarizes the CNR values for each mass size. Across all sizes, the CNR of DM was approximately 1.3–2 times higher than that of SM.

**TABLE 2:** Quantitative evaluation of contrast‐to‐noise ratio (CNR) for mass objects in the ACR 156 phantom.

Image	DM		SM	
Diameter (mm)	Average	SD	Average	SD
2.0	2.43	0.05	1.21	0.05
1.0	0.84	0.04	0.56	0.10
0.75	0.75	0.05	0.56	0.11
0.50	0.48	0.03	0.27	0.14

Contrast‐to‐noise ratios were calculated for each mass size using the same regions of interest as illustrated in Figure 3. The CNR values provide a quantitative assessment of mass detectability and complement the qualitative comparison shown in the phantom images. Across all mass sizes, DM exhibited CNR values approximately 1.3–2.0 times higher than those of SM.

#### 3.2.4 MTF

**FIGURE 4:**
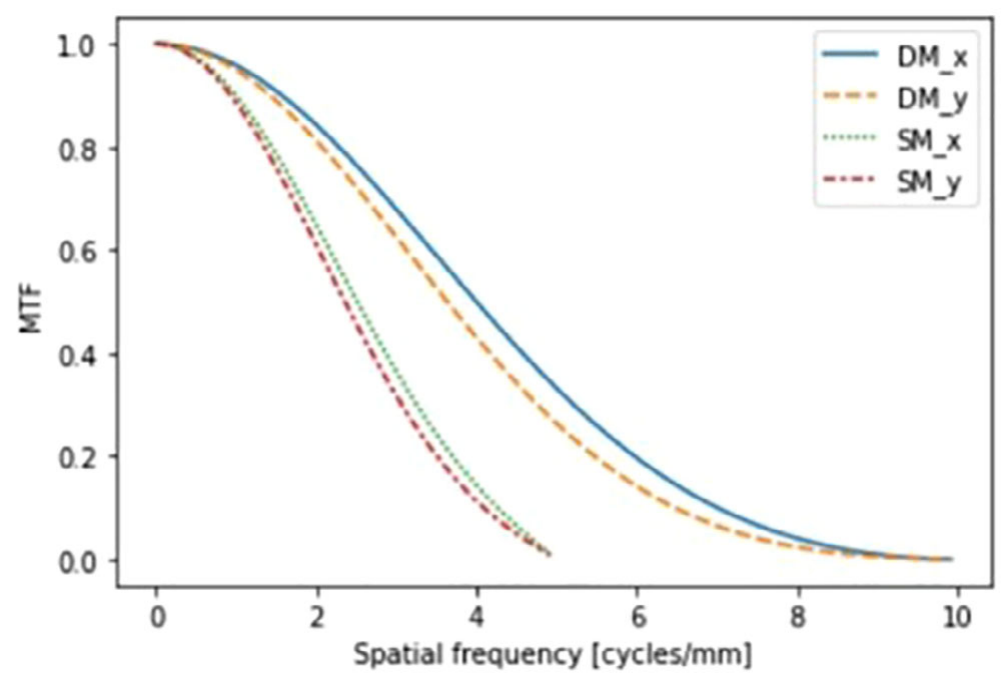
Modulation transfer functions (MTFs) for DM and SM. MTFs were derived using a 1‐mm tungsten edge phantom sandwiched between PMMA slabs to replicate clinical imaging conditions. A small slanted angle relative to the measurement direction was applied, and composite edge‐spread functions were generated from multiple line profiles. Due to the reduced contrast under these conditions, the composite edge‐spread function was modeled using an error function prior to differentiation to obtain the line spread function.

Figure 4 describes the MTF results. The pixel sizes were 50 µm for DM and 100 µm for SM, corresponding to Nyquist frequencies of 10 cycles/mm and five cycles/mm, respectively. The line plot types indicate the imaging modes and edge orientations. The MTF values were normalized to 0 cycles/mm. The spatial frequencies corresponding to MTF(f50) were 4.00, 3.63, 2.51, and 2.36 cycles/mm for DM_x, DM_y, SM_x, and SM_y, respectively. The spatial frequencies corresponding to MTF(f10) were 7.00, 6.47, 4.26, and 4.09 cycles/mm for DM_x, DM_y, SM_x, and SM_y, respectively.

#### 3.2.5 CD curve

**FIGURE 5:**
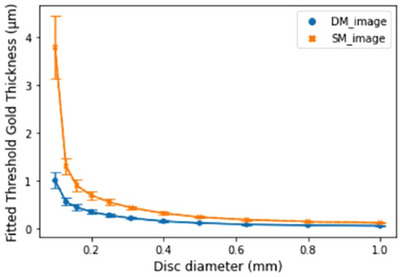
Contrast‐detail (CD) curves and inverse image quality figure ($IQF_{inv}$) for DM and SM obtained from CDMAM analysis. CD curves were generated using the EUREF CD analysis ImageJ plugin, with detection thresholds defined at a detection probability of 62.5%. For each contrast level, the threshold diameter was determined from the average results of 10 repeated acquisitions. The inverse image quality figure ($IQF_{inv}$) was calculated from the CD curves, where higher $IQF_{inv}$ values indicate better overall image quality, reflecting improved detectability of small and low‐contrast objects.

Figure 5 shows the CD curves obtained using the CDMAM phantom. The threshold was defined as a detection probability of 62.5%. Although no major differences were observed between DM and SM overall, SM exhibited higher threshold values for smaller disk diameters than DM, indicating a reduced detectability of finer structures. Consequently, the inverse image quality figures ($IQF_{inv})$, representing the area under the curve, were 134.9 for DM and 56.7 for SM, demonstrating superior performance of DM.

## 4 DISCUSSION

In this study, DM and SM images were compared using objective physical metrics, including SNR, NPS, CNR, MTF, and CD curves. Although a limited number of images were analyzed per condition, all measurements were performed using automatic exposure control to reflect routine clinical mammography practice, acknowledging that this results in variations in exposure parameters between imaging modes. Despite this variability, the acquisition conditions were stable within each imaging mode, allowing a reliable comparison of image quality metrics. The implications of these findings are discussed below:

### 4.1 SNR map and uniformity

Analysis of the SNR maps revealed that DM produced relatively uniform distributions, with higher SNR values concentrated centrally. In contrast, SM exhibited localized regions of elevated SNR in the lower portion of the image, with NUI values of 0.26 for DM and 0.54 for SM, indicating greater nonuniformity in SM. This nonuniformity is likely attributable to the reconstruction process of SM, particularly the projection synthesis and filtering algorithms.

Consistent with these findings, Yang et al. showed that differences in the noise characteristics—quantified via power‐spectrum analysis of breast parenchyma—substantially affect image appearance and may influence lesion detectability in s2D/DBT.^18^ Complementary phantom studies by Barca et al. further demonstrated that NPS‐ and CNR‐driven differences between SM and DM (and across SM reconstruction geometries) materially impact quantitative image quality. This supports the notion that noise properties play a dominant role in perceived image quality.^8^, ^19^ Similarly, prior work has shown that spatial variability in SNR can translate into variability in the detectability of small lesions, which is consistent with the observations of this study.^20^ Clinically, such nonuniformity in SNR may enhance or suppress local tissue density differences, potentially affecting the detectability of microcalcifications and small masses.^20^

The NUI used in this study represents a global measure of SNR variation within the analyzed region. While this metric is useful for characterizing overall image uniformity, previous studies have suggested that human visual perception is relatively insensitive to gradual global changes and is more strongly influenced by localized variations.^18^, ^19^ In the present study, although a dedicated local nonuniformity metric was not quantitatively evaluated, the SNR maps clearly demonstrated regionally localized variations in SM, particularly in the inferior portion of the image. These localized SNR variations may be more relevant to perceived image quality and lesion detectability than the global NUI value alone. Therefore, the use of a global uniformity metric alone is a limitation of this study. Future work should incorporate quantitative local nonuniformity measures, such as sliding‐window–based SNR variance or non‐parametric noise metrics, and ideally correlate them with observer performance or clinical detectability.

### 4.2 NPS‐based noise characteristics

The NPS results demonstrated that DM exhibited amplitudes approximately 1/100 of those of SM, indicating substantial noise suppression across both low‐ and high‐frequency regions. No clear periodic structures were observed in either modality; instead, the directional NPS analysis revealed non‐systematic fluctuations across spatial frequencies. While DM showed no significant directional dependence in the radial, *x*‐axis, or *y*‐axis analyses, SM exhibited significant directional variation, as reflected by the anisotropy indices.

These findings suggest that the noise structure in DM is finer and more uniform, whereas SM may incorporate direction‐dependent noise components introduced by the image reconstruction process. As noise characteristics directly affect perceived image graininess, these results support the superior performance of DM for the detection of low‐contrast lesions. Moreover, previous studies have reported that DM demonstrates lower noise levels than both conventional and synthesized mammography,^21^, ^22^, ^23^ and the present findings are consistent with this evidence.

### 4.3 CNR‐based detection performance

As indicated by the SNR analysis, the CNR values were influenced by the location of the background region. In this study, ROIs were defined to include mass objects, and the corresponding background was obtained from the same ROI after excluding the mass region.

The CNR evaluation demonstrated that DM consistently provided values 1.3–2 times higher than SM across all mass sizes. As CNR reflects the ability to discriminate lesions from the background, this difference suggests that DM offers superior lesion visibility. The pronounced differences observed for smaller masses are of particular clinical importance as they highlight the potential advantage of DM in early detection.

Moreover, DM tends to outperform synthetic images in terms of CNR,^24^, ^25^, ^26^ and the present results further support this evidence. Moreover, higher CNR values have been associated with improved ROC performance in observer studies,^27^ suggesting that physical image quality metrics, such as CNR, can serve as useful predictors of clinical diagnostic performance.

### 4.4 MTF‐based spatial resolution analysis

In this study, PMMA slabs were intentionally included during MTF acquisition to replicate clinical breast imaging conditions, where scatter, attenuation, and image noise substantially influence system performance. Since the purpose of this work was to compare the effective image quality of digital mammography and synthesized mammography as used clinically, evaluating the MTF under realistic imaging conditions was considered important.

The MTF analysis showed that DM (pixel size, 50 µm) achieved MTF(f50) and MTF(f10) values of approximately 4.0 and 7.0 cycles/mm, respectively, which were higher than those of SM (pixel size, 100 µm; 2.5 and 4.3 cycles/mm). These differences can be attributed not only to the variation in the Nyquist frequency because of the pixel size but also to the blurring introduced during the reconstruction process. The difference in MTF(f50) is particularly important, as it represents a critical indicator of the clinical resolution limit and supports the advantage of DM in detecting microcalcifications.

DM systems using direct‐conversion detectors maintain a high spatial resolution,^28^, ^29^, ^30^ and the present results are consistent with these findings. Moreover, the reduction in the MTF observed for SM may be attributable to the filtering effects associated with the synthesis algorithm, as previously suggested.^19^

### 4.5 CD curve and overall detectability

Analysis of the CDMAM phantom showed that the CD curves, with thresholds defined at 62.5% correct detection probability, revealed no major overall differences between DM and SM. However, SM exhibited higher thresholds for smaller disk diameters, indicating a reduced performance in the detection of fine structures. Consequently, the $IQF_{inv}$ values were 134.9 for DM and 56.7 for SM, demonstrating the marked advantage of DM in terms of overall detectability. These findings are consistent with the previously observed SNR uniformity and MTF results and highlight the limitations of synthetic images.

Moreover, synthetic 2D images perform worse than DM when evaluated using physical image quality metrics,^31^, ^32^ and the present results corroborate these findings.

Overall, DM outperformed SM for all evaluated parameters, including SNR uniformity, noise characteristics, CNR, MTF, and CD curve analysis. The present study focused exclusively on physical image quality evaluation and did not include dose measurements. Within this scope, DM remained superior for detecting fine structures and low‐contrast lesions. The view that SM is best used as a complementary rather than a replacement modality has been discussed by Melissa A. Durand,^31^ and our experimental results are consistent with that perspective.

This study has several limitations. First, the present study was conducted using phantoms with a uniform background, which does not fully represent the complex anatomical structure of the breast. In clinical imaging, lesion detectability is strongly influenced by structured anatomical background, where superimposed fibroglandular tissue may obscure lesions in DM. In contrast, synthesized 2D mammography derived from DBT may partially mitigate this masking effect by reducing tissue overlap. Therefore, image quality differences observed under uniform background conditions may not directly predict the relative diagnostic performance of DM and SM in anatomically structured backgrounds, and the present results should be interpreted with caution in a clinical context. Second, although standardized PMMA, ACR, and CDMAM phantoms were used to ensure reproducibility and objective comparison, these phantoms cannot replicate the heterogeneous texture and anatomical variability of real breast tissue. As a result, the influence of background complexity on lesion detectability could not be assessed in this study. Third, the evaluation was limited to physical image quality metrics and did not include observer‐based or clinical validation. While physical metrics provide valuable insight into fundamental image characteristics, they do not fully capture human visual perception or diagnostic decision‐making. Therefore, further observer studies and clinical investigations are required to clarify how the observed physical image quality differences translate into diagnostic performance. Finally, the image quality of synthesized mammography is known to be highly system‐ and algorithm‐dependent, and may be influenced by data‐driven or artificial intelligence–based reconstruction processes. Because phantom images are generally not included in algorithm training or optimization, phantom‐based evaluations may not fully reflect algorithm behavior in clinical images with complex anatomical backgrounds. Consequently, the findings of this study, obtained using a single commercial system, may not be directly generalizable to other vendors or reconstruction approaches.

## 5 CONCLUSIONS

In this study, we quantitatively compared the image quality characteristics of DM and SM using multiple physical metrics, including noise characteristics based on SNR and NPS, lesion detectability based on CNR, spatial resolution based on MTF, and overall detectability based on CD curves. DM demonstrated superior SNR uniformity, reduced low‐ and high‐frequency noise in the NPS, and higher CNR and MTF values compared with SM. CD curve analysis further confirmed superior detectability of fine structures in DM, particularly for smaller disk diameters.

In contrast, SM exhibited localized noise and nonuniformity, reflecting differences in image reconstruction characteristics. These findings demonstrate systematic differences in the physical image quality characteristics of DM and SM, suggesting that the two modalities may provide complementary information. However, the clinical implications of these physical image quality differences remain unclear. Further observer‐based studies and clinical image validation are required to clarify how these findings translate into diagnostic performance in clinical practice.

## AUTHOR CONTRIBUTIONS

Kazumi Sogabe conceived and designed the study, performed the experiments and data acquisition, conducted the data analysis, and drafted the manuscript.

Hiroki Saito (corresponding author) and Sho Maruyama contributed to image analysis.

Nao Koyama contributed to data acquisition.

All authors discussed the results, critically revised the manuscript, and approved the final version.

## FUNDING

This research received no specific grant from any funding agency in the public, commercial, or not‐for‐profit sectors.

## CONFLICT OF INTEREST STATEMENT

The authors declare no conflicts of interest.

## ETHICAL STATEMENT

This study involved only phantom experiments and did not involve human participants or animals. Therefore, ethical approval was not required.

## Data Availability

The data that support the findings of this study are available from the corresponding author upon reasonable request.
